# Neurobiological Implications of Parent–Child Emotional Availability: A Review

**DOI:** 10.3390/brainsci11081016

**Published:** 2021-07-30

**Authors:** Emma L. M. Clark, Yuqin Jiao, Karen Sandoval, Zeynep Biringen

**Affiliations:** Department of Human Development and Family Studies, Colorado State University, 1570 Campus Delivery, Fort Collins, CO 80523, USA; emma.clark@colostate.edu (E.L.M.C.); yuqin.jiao@colostate.edu (Y.J.); karenas@rams.colostate.edu (K.S.)

**Keywords:** emotional availability, biophysiological and neural mechanisms, parent–child healthy connections

## Abstract

Parental influences are important for a child’s behavior, overall adjustment, as well as cognitive/language development. New research is exploring how relationships with parents can influence a child’s neurobiological functioning and development. In this systematic review, our first aim is to describe how the caregiving environment influences these aspects of child development. The second and main aim is to review and recommend that the concept (and measurement) of “emotional availability” may provide a new window in this continued exploration. Emotional availability (EA) refers to the capacity of a dyad to share an emotionally healthy relationship. The EA Scales assess this construct using a multi-dimensional framework, with a method to measure the affect and behavior of both the child and adult partner (caregiver). In this review, we first provide an overview of child development research, with regards to stress physiology, neuroendocrine system, genetics and epigenetics, and brain mechanisms. We then summarize the results of specific EA research in these areas, and propose a theoretical model integrating these constructs. Finally, we offer areas for future research in this area.

## 1. Introduction

There is a great deal of scientific focus on the effect of early experience on a child’s neurobiological functioning and development. This line of research began with animal studies uncovering that maternal licking/grooming behavior in rodents is associated with less offspring anxiety and more optimal prefrontal cortical functioning [1]. In humans, evidence has accumulated that institutionally reared children have larger amygdala volumes as compared to those raised in the home setting [2]. The amount of time spent in the institutional environment has also been linked with amygdala volume [3]. Those children raised in the home setting, but under conditions of trauma, have also been a topic of focus. Trauma, such as child maltreatment exposure, can be harmful, in particular, when traumatic experiences happen at a young age, and such exposures may have long-term adverse effects on individuals. Specifically, studies of child maltreatment-exposed mothers have shown that they have a higher risk for adult psychopathological problems and low-quality parenting behaviors when compared to non-child-maltreatment-exposed mothers [4]. Early maltreatment has also been found to be associated with a lesser volume of the child’s corpus callosum, as well as lesser hippocampal volume in adulthood [5]. Further, maltreated children have been found to show enhanced neural responsiveness to “angry stimuli” such as facial expressions [6]. Additional work by Neukel, et al. [7] indicates that mothers with a history of early maltreatment are more likely to show effort in processing their own infant’s facial expressions of emotion, as evidenced by elevated activation of areas that are associated with the visual processing of faces (e.g., cuneus, middle temporal gyrus). These studies have established the impact of early adverse experiences on brain and physiological functions.

Outside of extreme adverse circumstances, such as child maltreatment, human research on the influence of the environment is only beginning. Learning how “normative” caregiving influences may be associated with children’s brain and physiological functioning is of vital importance. As such, Belsky and de Haan [8] wrote that research on the role of the environment on the brain is at the point of “the end of the beginning”, and that “work is now needed to determine whether and how variation in parenting in the normal range affects the brain development of children not exposed to extreme adversity” (p. 423).

While the evidence is scant, research indicates that parental warmth during interactions with one’s child at 18 and 24 months, as well as at 10 and 11 years, was associated with the child’s prefrontal cortical responding during a reward or loss, with maternal warmth serving an especially protective role in the case of boys who were exposed to maternal depression. Thus, even in the context of maternal depression, children (especially the boys) benefitted from the buffering role of maternal warmth [9].

Another important line of work is on attachment security in infancy. While a great deal of research has informed us of the importance of attachment in predicting behavioral and affective development, attention is only now turning towards the effect of attachment on brain and neural function. For example, Leblanc, et al. [10] reported that attachment security during infancy predicted whole-brain gray matter volume (assessed through structural magnetic resonance imaging) in the superior temporal sulcus dan gyrus, temporo-parietal junction and precentral gyrus, which are involved in social, cognitive, and emotional functioning in late childhood, at 10–11 years of age. However, there was no association with cortical thickness [10].

Maternal sensitivity has also been investigated to some extent, in relation to brain development in typically developing children. Higher maternal sensitivity has been found to be associated with greater subcortical volume in infants [11], but also to smaller hippocampal volume and smaller amygdala volume in infants [12]. In contrast, Kok, et al. [13] reported that early parental sensitivity (maternal and paternal combined) was actually not linked with hippocampal or amygdala volume, revealing some inconsistencies in specific brain regions in relation to this parenting construct. However, as would be expected, parental sensitivity was in fact associated with total brain volume in school-age children [12]. Moreover, as would be expected, the mother’s self-reported hostility, as well as observed aggression, were related to the child’s smaller total volume and cortical thinning, which are structures that are important for children’s social emotional/cognitive development, as well as emotion regulation [14]. Given the findings by Kok, et al. [15] that indicate a clearer link between brain volume and behavior for girls, at least in relation to prosocial behavior, additional research is needed to better understand the link between brain morphology and how this translates into behavior for each gender.

In addition to brain structures, research has also explored the link between the caregiving environment and neural activity, as measured by electroencephalography (EEG) or magnetoencephalography (or MEG). The EEG is a noninvasive means to assess the brain’s neural activity via the recording of electrical signals at the scalp. For an MEG, a Dewar containing multiple sensor coils are near, but not touching, the participant’s head, with recording of the brain’s magnetic activity. Perhaps the clearest example of this is a study by Hane and Fox [16], where they measured the observed caregiving environment (using a composite of the different Ainsworth scales, including sensitivity, acceptance, interference, degree of availability, pacing during feeding), and referred to this as maternal caregiving behavior (MCB). They found that low-quality MCB was associated with greater right frontal electroencephalographic asymmetry and fewer positive behaviors (e.g., fear, less joint attention). Further, they also found that lower MCB was associated with more negative observed infant affect. Levy, et al. [17] found a link between the caregiving environment (using a molecular index of mother–child synchronous interactions) and MEG recordings for that child many years later.

In addition to early deprivation and maternal behaviors, chronic stress has also been found to impact cognition, mental health, and brain structures [18]. Moreover, exposure to early life stress has been associated with increased reactivity to stress and cognitive deficits in adulthood [18]. When the brain detects a stressor, a physiological response is activated in the autonomic, neuroendocrine, metabolic, and immune systems. One system, the hypothalamus–pituitary–adrenal (HPA) axis, is an essential component of the stress system. Specifically, the hypothalamus releases corticotrophin-releasing hormone (CRH) and arginine vasopressin (AVP), which in turn triggers the pituitary gland’s release of adrenocorticotropic hormone (ACTH). This then leads to the adrenal cortex’s production of glucocorticoids. As specified, receptors for these steroids are expressed in the brain, and can regulate gene expression; therefore, glucocorticoids can influence brain functioning in the areas that regulate glucocorticoid release [18].

The HPA axis can be particularly useful in investigating emotion regulation in relation to neurobiology [19]. Past research has shown that parent–child interactions influence the child’s HPA axis activity, especially during infancy, in which factors such as maternal socioeconomic status and depressive state are associated with better regulation of the HPA axis to everyday stressors [20]. Children who experience a healthy caregiving environment are better able to regulate their stress, as demonstrated through the HPA axis [21]. For example, parent–child synchrony, measured through moment-to-moment analyses, has been found to be related to more effective child stress regulation [22].

Research into genetics and attachment has largely focused on oxytocin, a hormone that is key to bond formation and social reciprocity, in which it is theorized that an individual’s attachment bonds are underpinned by the oxytocinergic system. Previous research has shown that maternal oxytocin levels, in response to play with their infants, were associated with the activation of the hypothalamus/pituitary region and the right ventral striatum [23]. Moreover, individual genetic differences between DNA sequences influence how genes are expressed, and how, in turn, their respective encoded proteins (such as oxytocin) function. Infant early caregiving experiences may be partially influenced by parental oxytocin levels, and such oxytocin levels may transfer cross-generationally through caregiving and maternal genetics, further emphasizing the significance of promoting healthy parenting [21].

Environmental experiences that occur before and after birth can cause chemical modifications to specific genes, influencing how much and when they are expressed [24,25]. This phenomenon is known as epigenetics. Epigenetic changes are phenotypical changes in gene expression to portions of the DNA, not alteration of the genetic sequence itself, which are often experience-driven [19]. Experiences that occur early in life, when the brain undergoes tremendous changes, may cause epigenetic changes that influence how specific genes instruct the body to grow biologically, behaviorally, and neurologically; therefore, it is particularly important to cultivate supportive, quality parent–child relationships during these years [26,27,28].

As noted above, studies on early deprivation, trauma, as well as normative variations in parenting, indicate a significant link with a child’s neurobiological functioning. However, studies are limited to broad constructs such as “sensitivity”, with some studies including many other qualities in this broad construct, whereas others do not, and yet they seem to all be referred to as sensitivity. Further, the child’s side of the parent–child equation appears to be missing, except when child attachment security has been assessed.

The topic area of “emotional availability” is seeing a growing interest in neurobiological functioning [29,30]. One reason for this may be that parental qualities such as sensitivity, structuring, nonintrusiveness, and nonhostility can be investigated using the same system. Qualities such as maternal warmth and maternal sensitivity are obviously related to one another, but the actual measurement of constructs is also important. Note the eloquent piece that “measures matter” by Bohr, et al. [31]. Just because measures sound similar to one another, it does not mean that they are the same. In addition to the parental side of emotional availability, the EA System places great emphasis on the child’s side of emotional availability (child responsiveness to the adult and child involvement of the adult). The child’s side has been virtually ignored in the field of affective neuroscience, except for some minimal investigation of the child’s attachment security. A system that includes both the parental and child sides, as well as attachment security/bonding, would appear to be useful.

In recent decades, emotional availability (EA) has been a topic of interest for developmental researchers, and has been widely studied in the scientific and scholarly literature [30]. The current conceptualization of emotional availability encompasses the following two aspects: (1) how parental EA influences the child’s development and responses, and (2) how child EA influences the parent’s feelings of value and ability to provide appropriate care for their child [30]. In 2014, Biringen, Derscheid, Vliegen, Closson and Easterbrooks [30] conducted a comprehensive review of the published EA literature that emphasized the need to include at-risk populations with mental health disorders and/or disabilities, and to increase EA measures in intervention work [30]. Since the 2014 review, EA researchers have indeed increased their focus on at-risk populations, and have provided valuable information on a variety of disorders (e.g., depression, substance use disorders, personality disorders, and schizophrenia), as well as a new measure of prenatal EA [32,33]), which can be assessed both as self-report as well as observation [34]. Moreover, past research has suggested that environmental experiences and behavioral practices can influence brain structure and function, and may be shaped by genetic co-evolutionary mechanisms [26]. However, there has been limited research focused on understanding the underlying biological and neural mechanisms of EA on parent–child interactive behaviors and development. Moreover, cultivating secure, emotionally available relationships, along with developing appropriate stress regulation skills, are important aspects to a child’s healthy brain development [24,35,36]. As the dyadic nature of EA focuses both on the parent as well as the child, with the goal of improving the parent–child relationship, addressing physiological cascades that are influenced by improved EA would dramatically add to our understanding of affective neuroscience, as well as the intergenerational genetic transmission of emotional connections. Thus, a review of stress physiology and the neuroendocrine system, genetic and epigenetic influences, and brain mechanisms associated with EA, is warranted.

Our first aim of this review is to explore and describe how the caregiving environment influences a child’s neurobiological functioning and development. Our second, and primary aim, is to review and recommend the concept (and measurement) of EA as an innovative methodology that will add important information into furthering this field’s research. Our review unfolds as follows. First, we provide an overview of the EA framework. Second, we review neurobiological factors that are related to EA, with regards to stress physiology and the neuroendocrine system, genetics and epigenetics, and brain mechanisms. We present a theoretical model beginning with prenatal EA and postnatal EA. We then discuss implications of improving the health and wellbeing of parent–child relationships, as well as the potential for informing future interventions.

### 1.1. The EA Framework

The EA framework comprises the following three operationalized measures that allow researchers to evaluate how each member of the parent–child dyad affects the other member: (1) observational EA Scales, (2) observational EA zones, and (3) EA Self-report [30]. The observational EA measures are currently on their 4th edition [30].

### 1.2. The EA Scales

The EA Scales specifically differentiate adult and child experiences and perspectives for use in research and clinical practice. Specifically, there are the following four dyadic EA Scales for the parent: (1) sensitivity, (2) structuring, (3) nonintrusiveness, and (4) nonhostility. Further, there are the following two dyadic EA Scales for the child: (1) responsiveness and (2) involvement [30].

### 1.3. Adult EA Scales

Adult sensitivity refers to the caregiver’s ability to attend to the child’s emotional needs and behavioral cues; this scale is dyadic, and is dependent on the relationship with, and the response of, the child. Adult structuring is the ability of the caregiver to support the child’s activities and autonomy through guidance, scaffolding, or mentorship. Adult nonintrusiveness refers to the caregiver’s lack of interference with the child’s behaviors through over-direction, over-stimulation, interference, or over-protection. Adult nonhostility is the absence of hostile acts that parents may intentionally or unintentionally directly target towards their children. A parent’s behavior is rated and viewed in a manner that is dependent on the way the child responds [30].

### 1.4. Child EA Scales

Child responsiveness refers to the responsiveness of the child to the parent’s bids. Child involvement refers to the child’s ability and desire to involve the caregiver in their play or activities [30].

### 1.5. EA Zones

The EA System now also includes EA zones for the parent and the child, which align with the four attachment styles of secure, insecure-avoidant, insecure-resistant, and disorganized [37]. These zones provide a dimensional view of “emotional attachment” (on a 100-point scale), in addition to providing information on the four attachment categories. Moreover, the EA zones have been validated in terms of secure/insecure attachment in different studies, using the strange situation procedure, the attachment Q-set, and the adult attachment interview [38]. Specifically, the EA zones include the following four different zones of emotional attachment of the parent and child: (1) emotionally available, (2) complicated, (3) detached, and (4) problematic/disturbed [29].

The zone placement for the parent is predominantly dependent on the parent’s sensitivity score, and the zone placement for the child relies on the child’s responsiveness score, with higher scores in their respective scale being associated with being in the “emotionally available” zone. The mid-range scores of child responsiveness refer to a “complicated” emotional attachment, and low scores, below the mid-range of child responsiveness, are referred to as being “detached”. Finally, “problematic/disturbed/traumatized or traumatizing” is the lowest zone [29]. The additional EA dimensions (structuring, nonintrusiveness, nonhostility, and child involvement) are further important when assessing the relationship, and may be used to place an individual into the lowest zone, but sensitivity and child responsiveness scales “kickstart” the EA zones placement decision [29]. Most importantly, the EA zones provide an attachment perspective from both the parental side as well as the child side, which extends traditional attachment research in which observing only one perspective is a common practice, with the parent and child having their own distinct zone, which may or may not be the same, suggesting that the attachment of each may be different. Additionally, within each zone, there is the option to rate some higher than others, with the inclusion of a dimensional way of looking at a zone, as for example, low on emotional availability, but nonetheless in the emotionally available zone, or high on complicated, but still in the complicated zone rather than one zone up, which would be the emotionally available zone. Finally, the EA zones include a description of emotions as they impact attachment; thus, they are zones of “*emotional* attachment”.

### 1.6. EA Self-Report (EA-SR)

The EA Self-Report (EA-SR) is a parent-reported measure that is inspired by the observational EA Scales and correlates with the observational EA dimensions, to a moderate extent [39,40]. The EA-SR consists of 36 statements that are rated on a 5-point Likert scale, from “1” (*Almost Never*) to “5” (*Always*), which assesses the parent’s perception of the parent–child relationship, and the EA between themselves and their child. Parents are asked how characteristic each statement is about their relationship with their child, with questions such as, “My child clearly enjoys being together with me”. At this time, and with only one study that conducted a factor analysis [41], the EA-SR items load on to the following five factors: (1) mutual attunement, (2) affect quality, (3) dyad interactions, (4) intrusiveness, and (5) hostility. The EA-SR has demonstrated good internal reliability and construct validity compared with the observational EA Scales [40,41]; however, it is important to note that the link between the observational EA Scales and the EA-SR is moderate at best [41]. The EA-SR is now included in the overall EA System, but it is still recommended to use the EA Scales or EA zones rather than the EA-SR, if the situation permits. The EA-SR is referred to as “self-reported EA” rather than EA, since the latter refers to the observational system.

### 1.7. Prenatal EA

In addition, a new measure of EA has been developed—assessing prenatal emotional availability (pre-EA), which has been validated in Finland [34]. In the pre-EA, the mother is videotaped while performing activities with her fetus. Unlike the EA (postpartum to 14 years, soon to be extended to 17–19 years) that is scored on six scales (described above), the pre-EA only includes the following two maternal scales: maternal sensitivity and nonhostility. Assessment of the pre-EA focuses on the affective and behavioral cues of the mother. The pre-EA measure has similarities to previous work on prenatal attachment; however, the pre-EA is an observational measure about the mother in interaction with her unborn baby, in contrast to prenatal attachment measures that are typically self-reported [32,33]. As numerous studies have examined postpartum or early postnatal EA to two months of age, it would also be a beneficial addition to incorporate further study of EA during pregnancy [42,43,44].

## 2. Methods

**Figure 1:**
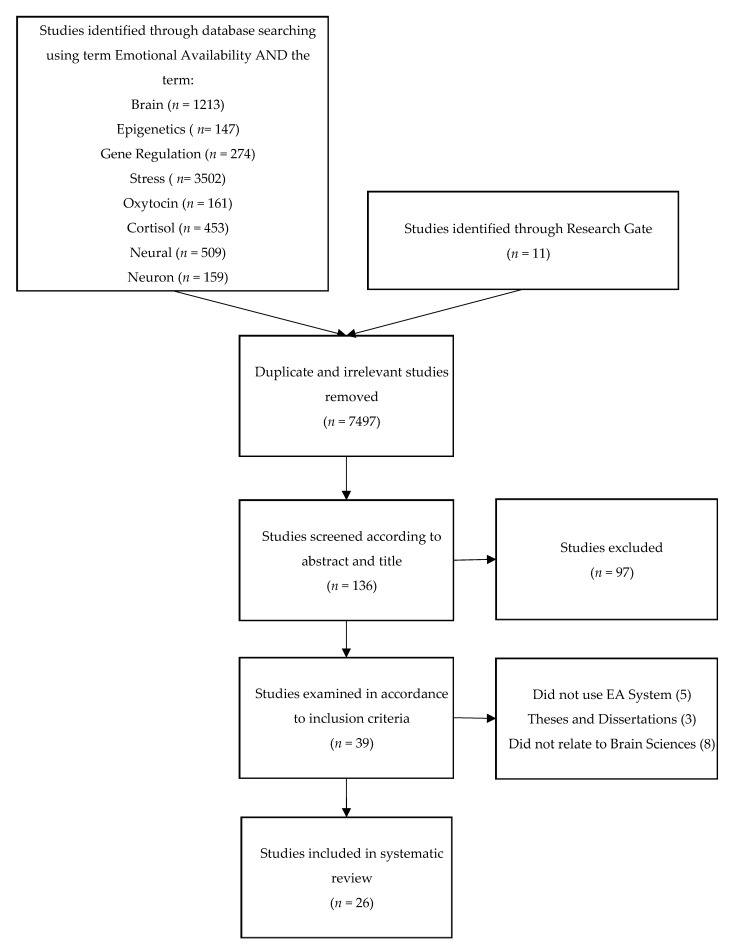
Literature search process and study selection flow.

The preferred reporting items for systematic reviews and meta-analyses (PRISMA) statement was designed to transparently report the reasoning, methods and results discovered by the authors, and improve the quality of reporting in systematic reviews through an evidence-based 27-item checklist [45]. This systematic review was conducted in adherence to the guidelines and checklist. A visual representation of this review’s search and selection criteria is depicted in Figure 1.

### 2.1. Literature Search

The journal articles included in this review were compiled through online searches, utilizing the entirety of the Colorado State University (CSU) online library as well as Google Scholar to access 374 journal databases. A general search of the key terms scoured all 374 databases in the online library, which included databases such as PsychInfo and Medline. Searching for studies in this way assured that all studies using an EA system, and those that were published in a database made available through the CSU online library, were included and found. Thus, the literature search was conducted by searching for terms in the general online library, rather than searching each database individually.

Key terms that were used in order to screen for articles focusing on neurobiological correlates in relation to EA included, “EA”, “emotional availability”, “brain”, “genetics”, “epigenetics”, “biological”, “gene regulation”, “stress”, “oxytocin”, “cortisol”, “neuroscience”, “neural”, “neural”, “neurons”, “physiological”, and “executive functioning”. Other journal articles were located through citation notifications of author Biringen through Research Gate.

### 2.2. Study Selection

Studies were selected a priori using the following inclusion criteria: (1) utilized at least one of the measures in the EA System (EA Scales, EA-SR, or EA zones) as a measure; (2) directly included the measure in the reported results; and (3) related the measure to the neurobiological sciences. The exclusion criteria was as follows: (1) studies not using the EA System; (2) did not report on one of the EA measures in the results; (3) did not relate to neurobiological sciences; and (4) were theses or dissertations, and therefore, not peer reviewed. Thus, the authors chose to include any study using any part of the EA System and focusing on neurobiology, and this was conducted on an a prior basis, given that we wanted to focus on the literature on EA specifically.

### 2.3. Data Extraction

We extracted and condensed the following information: (1) target population, including age, sample size, and sample characteristics; (2) the EA measure used in the study and how it was scored; (3) any applicable additional variables (e.g., cortisol sampling or brain imaging); (4) EA reliability scoring, where the observational system is used; and (5) key results. The tables which show this in detail are at the end of the document.

### 2.4. Quality Evaluation

The quality of the included articles was rated in line with guidelines by the *Cochrane Handbook for Systematic Reviews of Interventions* [46].

## 3. Results

The literature search yielded 7633 articles. After excluding duplicates and studies that did not meet the inclusion criteria, 26 articles in total were included in this systematic review.

### 3.1. Stress Physiology, the Neuroendocrine System, and EA

#### 3.1.1. Stress Physiology

Throughout the existing literature on EA, multiple studies have found a connection between this aspect of parent–child relationships and child stress physiology. In one study using the EA Scales to measure parent sensitivity, structuring, nonintrusiveness, and nonhostility, Kertes, et al. [47] used a principal component analysis to combine these four measures of observed parental EA into a factor score that was labeled “Parenting Quality”. Their results suggested that sensitive, supportive parenting that was indicated by a higher score of parenting quality, may act as a buffer of the HPA axis stress response in infants, toddlers, and preschoolers [47].

This result is also consistent in samples of children with an immigrant background, in which those who experience higher EA scores on sensitivity, structuring, nonintrusiveness, child responsiveness, and child involvement, demonstrated more effective stress regulation at kindergarten entry [48]. Rickmeyer, Lebiger-Vogel and Leuzinger-Bohleber [48] reported that in immigrant households (immigrants from some 17 different cultures in Germany), the children’s cortisol levels were higher after kindergarten entry than before; lower EA before kindergarten entry was associated with a rise in hair cortisol concentrations (HCC) (negative correlation); the children with lower mother intrusiveness and higher child responsiveness showed lower cortisol increases.

Similar to the above, but using the EA zones, which is a measure of “emotional attachment”, Senehi, et al. [49] investigated HCCs in relation to adverse childhood experiences (ACEs) and EA. They found that only in the low EA zones (which was defined as anything that is not in the emotionally available zone), there was a link between ACEs and HCCs. Thus, for children with parental history of multiple ACEs, those children of parents with higher EA had lower HCC compared to those children of parents with lower EA, indicating the buffering role of EA [49].

Similarly, Philbrook and Teti [50] examined EA at bedtime in relation to the stress reactivity of the infant. Infants with mothers who were scored as more emotionally available (measured through creating a composite maternal EA score with the EA Scales 3rd edition) at bedtime, showed less infant distress and more sleep (coded through videosomonography, where the infant’s sleep–wake states were objectively assessed), as well as lower infant salivary cortisol levels, as compared to those with lower EA mothers [50].

Another study by Ruttle, et al. [51] used the EA Scales from the EA 2nd edition to create a variable capturing dyadic behavioral sensitivity through a principal component factor analysis with sensitivity, structuring, nonhostility, child responsiveness, and child involvement, and examined the synchronization (or “attunement”) of the hypothalamic–pituitary–adrenal (HPA) axis between the mother and child. The results of this study indicated that such cortisol attunement only occurred during significant periods of challenge, and only in behaviorally sensitive mother–child dyads [51].

Several studies have linked EA and stress, but few have looked at the relationship between EA, stress, and stress mitigating factors. One well-researched stress contributing factor is the impact of low socioeconomic status (SES). Tarullo, et al. [52] examined the link between SES and cortisol functioning in early childhood, using a multidimensional approach to assess SES and cortisol concentration. Measuring income-to-need, parent education, occupational prestige, neighborhood risk, food insecurity, and household chaos allowed the researchers to look at the underlying SES factors that play a role in cortisol functioning. Additionally, researchers used both hair cortisol concentrations (HCC) and salivary cortisol concentrations (SCC) to measure how SES risks may contribute to parenting. The EA sensitivity scale was used to measure parenting. A relation between higher child HCC and higher parent HCC, as well as lower SES (indicated by parent education, occupational prestige, and greater food insecurity), was established; however, lower parental sensitivity was only related to higher child HCC in the 3.5-year-old group compared to the 12-month-old group. Parental sensitivity was unrelated to the infant cortisol measures. Despite finding a connection between parental sensitivity and HCC, parental sensitivity was not found to mediate links between SES risks and HCC in the 12-month-old group or 3.5-year-old group [52]. These results suggest that the interactions between parent sensitivity and child hair cortisol are likely to be complex, and it would therefore be beneficial for researchers to utilize the entirety of the EA Scales in order to more accurately observe how SES risks may play a role in parenting and child cortisol functioning.

#### 3.1.2. Skin Conductance and Heart Rate Variability

Other methods to assess stress physiology have been heart rate variability and skin conductance. An interesting study by Gilissen, et al. [53] reported that temperamentally fearful children showed less stress reactivity to fear-evoking film clips if they were being raised by higher EA mothers, but this link was not found for those who were not temperamentally fearful. These findings support the importance of the rearing environment, especially for this group of more susceptible children [53].

EA research, to this point, has consistently found a connection between EA and stress; therefore, it is important to further investigate rigorous, appropriate measures of stress (e.g., hair and saliva cortisol), along with the potential adverse effects of stress, in order to best inform prevention and intervention research. Moreover, it is recommended to use the entire EA System (i.e., sensitivity, structuring, nonintrusiveness, nonhostility, child responsiveness, and child involvement) when assessing for EA, rather than a select number of dimensions, such as only sensitivity or parenting scales, as each dimension has the potential to add unique information to the research.

#### 3.1.3. Testosterone

Of course, father–child interactions are the place to study the link between basal testosterone levels and EA. van der Pol, et al. [54] studied this link in the context of self-regulation, and found that higher testosterone levels in the evening were associated with less respect for the child’s autonomy, but only if the father also had problems with self-control. For fathers who were generally well regulated, higher testosterone in the evening was associated with greater sensitivity [54].

#### 3.1.4. Oxytocin

Oxytocin is another important neurotransmitter to include when discussing the neuroendocrine system and caregiving. MacKinnon, et al. [55] examined the influences of oxytocin during the end of pregnancy and postpartum on maternal behaviors, using the EA scales. Researchers found that oxytocin that was measured at 32–34 weeks gestation was indirectly associated with greater maternal structuring and fewer intrusive maternal behaviors at 2–3 years postpartum, through the mothers’ theory of mind (one’s ability to understand and take into account another individual’s mental state) [56]. The results of this follow-up study indicated that one’s theory of mind ability may represent a social cognitive mechanism linking endogenous maternal oxytocin and maternal caregiving [55].

In a study by Naber, et al. [57], the influences of oxytocin on father–child interactions were investigated using intranasal oxytocin administration and the EA Scales. Specifically, fathers who received the intranasal oxytocin were able to stimulate their child’s exploration and autonomy more effectively compared to the placebo condition. Moreover, fathers in the oxytocin condition tended to demonstrate less hostility compared to the placebo condition. However, the oxytocin condition did not lead to increases in sensitive interactions. This is an intriguing example of the transactional nature of parenting and the neuroendocrine system, with changes in oxytocin levels inducing changes in caregiving, and specific parenting behaviors leading to changes in oxytocin levels. These findings suggest that oxytocin may be an important mechanism in parenting, by which socially relevant cues in the dyad activate dopaminergic pathways and can positively reinforce responsive parenting behaviors in a positive feedback loop [57].

### 3.2. Genetics and Epigenetics

#### 3.2.1. Genetics

In a study by Reichl, et al. [58], researchers examined three variants of the OXTR gene on parenting behavior as well as plasma oxytocin. The EA Scales were used to measure one dimension of EA, which was maternal sensitivity. One of the OXTR gene variants, rs53576, did demonstrate a direct effect on maternal sensitivity; however, the other two gene variants, rs2254298 and rs104778, did not have this direct effect. Moreover, another of the OXTR variants (a rare variant A of the rs2254298) did moderate the relation between mothers’ experience of childhood abuse and her sensitivity, suggesting that this variant may act as a protective factor between mothers’ childhood abuse and their parenting sensitivity. It was also found that mothers’ childhood abuse was related to lower maternal sensitivity and to a higher child abuse potential, but only among the mothers who were homozygous for the common allele (GG) compared to those who were not homozygous. Finally, the G allele of the OXTR variant rs2254298 was associated with elevated plasma oxytocin levels [58]. Overall, developing a more comprehensive understanding of the interconnections between genetic level variations and full assessments of parent and child emotional variability would provide the field with information that could be used to provide predictions of maternal behavior.

As the EA System assesses parent–child relationship quality from both parent and child perspectives, the use of EA in assessing qualities would provide essential information about potentially modifiable aspects of the relationship that may influence the oxytocinergic system. Moreover, the incorporation of EA in genetics research would further provide information needed for targeted, preventative interventions, designed to promote healthy parent–child relationships.

#### 3.2.2. Epigenetics

Promoting healthy parenting may help promote the healthy intergenerational transmission of genetic material. Lecompte, et al. [59] examined mother–child interactions, preschool-aged child’s controlling-attachment behaviors, and DNA methylation of the oxytocin receptor (OXTR) gene in the child. Mother–child interactions were assessed by a 5-min free play and coded with the EA Scales, child attachment behaviors were measured by the separation–reunion procedure, and children’s DNA methylation of the OXTR gene was obtained by buccal swab. They found that lower maternal sensitivity was predictive of a more child controlling-caregiving form of attachment and less structuring was predictive of controlling-punitive attachment. Maternal structuring was associated with the hypomethylation of the OXTR gene (associated with caregiving) [59].

Further, a different study by Lewis, et al. [60] investigated how epigenetic processes may be a mechanism by which early social experiences may shape immune functioning and general health. This particular study investigated the differences in monozygotic twins, to assess how their primary caregiver’s emotional availability with each of them, measured by the EA-SR over time, predicted their immune gene methylation at 8 years. They found that maternal self-rated emotional availability at a child age of 1 year was related to the methylation of multiple inflammation genes in the monozygotic twins at 8 years of age. Further, twin pairs who were *discordant* in health had *more* differences in their methylation of inflammation genes, and twin pairs who were discordant in health had larger differences in EA with their mothers, compared to twin pairs who were more concordant in health. However, note that the EA findings are based on self-reports, such that mothers may have been rating these relationships based on the child’s health. Alternatively, there may have been real differences in EA in these mother–twin relationships. It would be important to investigate EA in an observational context [60].

### 3.3. Brain Mechanisms

EA research has largely focused on behavioral factors. Much less is known about the brain mechanisms that are associated with EA. Existing studies have shown the importance of parent–child interactions in children and parents’ brain development. To our best knowledge, there are 13 studies in total linking brain mechanisms to different EA constructs.

#### 3.3.1. Neglectful Mothers

Neglectful parenting is considered an adverse childhood experience (ACE) for children, and can have negative long-term impacts on children’s health and development [61]. Moreover, neglectful mothers tend to show lower levels of emotion expression and less reactivity to their child’s emotional signals during mother–child interactions [62].

Two recent studies investigated the EA Scales in relation to structural brain health--neglectful and non-neglectful mothers. In the first study, Rodrigo, et al. [63] studied the white and gray matter volumes of 25 neglectful (NM) and 23 non-neglectful or control mothers (CM), and related this to mother–child interactions. They performed structural MRI with voxel-based morphometry to examine brain volumes, and coded a gameplay task of the mother and child with the six adult and child EA Scales. Compared to CM mothers, NM mothers showed smaller gray matter volume in the right insula, anterior/middle cingulate, and right inferior frontal gyrus, as well as less white matter volume in the bilateral frontal regions. Specifically, areas related to empathy, regulatory control, and reactions to infant pain and distress, as well as the mirror neuron system, which enables the parent to attune at an intuitive level, appear to be affected in NM mothers [63].

Additionally, in a structural MRI study by León, et al. [64], cortical thickness and surface area were examined for 45 mothers (24 NM and 21 CM mothers), and examined in relation to EA as well as alexithymia (difficulty identifying and expressing emotions) traits. Compared to the CM mothers, the NM mothers showed thinner cortical thickness in the right rostral middle frontal gyrus and orbitofrontal cortex, and greater surface area in the right lingual and lateral occipital cortices. Further, although the direct path between the right rostral middle frontal gyrus and EA was significant, and remained significant even when the alexithymia traits were subtracted, nonetheless, such alexithymia traits reduced the strength of the relation. Thus, the frontal areas of the brain have important implications in the bidirectional attunement of mother–child interactions. The results of this study suggest the importance of further considering the association of cortical thickness and EA among NM mothers, and providing insights into the understanding of the neural mechanism behind neglectful mothers’ behaviors [64].

In sum, these two studies, examining the effect of neglectful experiences on the mother’s own brain volume and functionality, may help the design of intervention programs that seek to improve maternal response to infant distress cues and signals, as well as assess interventions that can create the context for modifications of the brain.

#### 3.3.2. Child Maltreatment-Exposed Mothers

Neuroimaging studies of linking child maltreatment-exposed (CME) mothers to EA constructs are sparse. We identified five studies here in this review. First, Rodrigo, et al. [65] studied the white matter alterations in neglectful mothers who had experienced maltreatment in childhood. They used the six EA Scales to access the quality of the emotional exchanges of the parent and child in 22 neglectful and 22 control mothers with children who are younger than 5 years old. They performed functional MRI to estimate six elements of diffusion tensors and associated fractional anisotropy and mean diffusivity, and tracked the white matter connectivity between the frontal, temporal, and occipital regions of the brain. Overall, the two groups of mothers differed by the streamlines of the inferior fronto-temporo-occipital connectivity, in particular, neglectful mothers showed fewer streamlines in this region that is known for the involvement of the face-processing network. Furthermore, they found fewer streamlines, especially in the right inferior longitudinal fasciculus tract (IFL-R), and observed that this was predictive of lower EA scores. Neglectful mothers showed lower sensitivity to the child’s cues and demands, as compared to non-neglectful mothers. These white matter alterations that form atypical fronto-temporo-occipital patterns, have an impact on the interactions indexed by EA between a mother and her child; thus, greater volume in the ILF-R was also predictive of positive mother–child interactions [65].

In addition, Olsavsky, et al. [66] recruited 26 CME and 19 NE mothers to participate in a mother–infant social communication study, and measured amygdala reactivity from functional MRI during an adult–infant face task. They used a 15 min mother–infant free play to code the four maternal EA scales (sensitivity, structuring, nonhostility, and nonintrusiveness), with a focus on maternal sensitivity. They found that CME mothers showed higher amygdala reactivity to infant faces, and this was associated with greater maternal sensitivity during mother–infant interactions, but such an effect was not observed during adult face tasks. The study results suggest that childhood maltreatment could affect maternal neural processing of social cues of infants [66].

Moreover, Olsavsky, et al. [67] focused on mothers with CME and how such experiences influence their responses to children’s cues. Specifically, they collected information on mothers’ CME through the risky families questionnaire (using the physical abuse, verbal abuse, and witnessed domestic violence questions), and recorded (by functional MRI) amygdala activation and connectivity to motor planning and empathy regions in the brain in response to their child’s cry vs. other stimuli (other infant’s cry and white noise). The researchers found that mothers reporting higher levels of CME showed higher amygdala activation to their own baby’s distress cries when compared to other infant’s distress cries or white noise. Thus, the connectivity between the amygdala and the areas associated with the motor planning and empathy regions of the brain (middle frontal gyrus) are heightened with greater mother-reported CME. Moreover, higher amygdala activation and heightened connectivity with prefrontal areas were positively associated with maternal nonintrusiveness (curiously, with no differences documented for maternal sensitivity and hostility). Those who reported greater CME may have been showing a maternal adaptive response (i.e., an increased sense of protection over the infant) in the context of their infant’s distress signals (vs. non-distress signals) [67]. 

Neukel, et al. [68] investigated the caregiving behavior of mothers with early life maltreatment (ELM) history (physical and/or sexual abuse or neglect), and used the maternal sensitivity scale of EA to investigate the effect of ELM on interactions in the next generation. They invited 47 mothers (22 ELM and 25 control mothers, who had none of the above indicators of maltreatment in their early life history) to interact with their children (7–11 years old), and collected their functional MRI during real-life interactions as well as imagined conflictual and pleasant interactions. The findings indicated that the ELM mothers were less sensitive than the control mothers in their real-life interactions, but while imagining conflictual interactions with their own child, they showed increased activation in the amygdala, insula, and hippocampus, which are known to be involved in emotion-processing, with early experiences of trauma likely sensitizing these mothers to their child’s distress communications, but not their pleasant communications. Thus, ELM mothers appear to be highly vigilant and responsive to negative interactions with their own child, but lack proper responsiveness and sensitivity during imagined pleasant or real-life mother–child interactions [68].

Mielke, et al. [69] investigated the relation between maternal sensitivity and “the empathic brain” in 25 mothers with, and 28 mothers without, an ELM history of physical and/or sexual abuse. Mother–child pairs were invited to engage in a 15-min free play and a 6-min problem-solving task, and their interactions were rated by EA Scales (only the maternal sensitivity scale). A voxel-based morphometry method was used to collect the structural MRI of the mothers’ brains. Overall, compared to mothers without ELM, mothers with ELM were less sensitive when interacting with their own child, and yet there was a huge range in sensitivity in both the groups. Importantly, for mothers with ELM, a positive relation was found between their maternal sensitivity and their self-reported cognitive empathy, as well as grey matter volume (in the superior temporal sulcus, temporal poles), which are core regions of the “cognitive empathy” network. For mothers in the control group, on the other hand, their maternal sensitivity was related to grey matter volume (in the anterior insula), which is a core region of the “emotional or intuitive empathy” network. The authors concluded that mothers with ELM compensate for their emotional deficits by recruiting more brain regions involved in cognitive empathy when engaging in the gameplay [69].

#### 3.3.3. Normative Samples

Six studies examine brain mechanisms in normative samples.

A study by Firk, et al. [70] focused on the amygdala response to infant crying, but in a normative sample. Firk, Dahmen, Lehmann, Herpertz-Dahlmann and Konrad [70] investigated 26 mothers without any genetic syndrome or severe disease with their full-term infants. The quality of the mother–infant interactions was assessed by a 12-min free-play coded by using EA Scales (maternal sensitivity, maternal instructing, maternal nonhostility, and maternal nonintrusiveness) and a 6-min still-face task. Functional MRI was used to collect the mothers’ amygdala activities while presenting sound stimuli (own infant cry, other infant cry, or control sound). The authors reported that maternal sensitivity and maternal nonhostility were negatively associated with amygdala activation during mother–infant interactions. Furthermore, downregulation was shown in the amygdala when mothers practiced self-distraction as an emotion regulation method. Therefore, it was suggested that enhanced activation in the amygdala, in response to infant crying, might be related to less sensitive and more hostile maternal behaviors; fortunately, self-distraction is an effective method in decreasing the emotional response to infant crying [70].

Another study looking at families who are socioeconomically disadvantaged (again in a normative sample), examined amygdala responses, using fMRI, to infant emotional expressions (positive, negative, and neutral faces). Compared to the previous study that took a multidimensional approach to SES, Kim, et al. [71] only looked at the income-to-needs (ITN) ratio, but they did use the entirety of the EA Scales and further only looked at maternal sensitivity and nonintrusiveness. They found that mothers did have an elevated amygdala response to negative infant faces and less neural responses to positive infant faces, and this was associated with the mothers’ parenting behaviors and ITN ratio. Their indirect effect finding suggested that a lower ITN ratio was linked to elevated amygdala responses to negative infant faces, and this was further associated with lower nonintrusiveness (meaning the mother was observed to be more intrusive) during the interaction. Similarly to the study by Tarullo, Tuladhar, Kao, Drury and Meyer [52], there was no effect for maternal sensitivity [71]. This finding has huge implications for future interventions for low-income mothers, who may be more vulnerable to altered neural processing of emotional expressions, and calls for more research on the links between SES, stress physiology, neural processing, and all of the EA Scales. For example, it would be interesting to see how household chaos (a SES index) may be related to, or impact, the EA scale structuring, due to unstable routines. Insight into the different EA dimensions, including child responsiveness and involvement, may provide valuable information on the relation between cortisol functioning and low SES, and inform future child intervention and prevention efforts focused on low-income children and youth.

In addition to the aforementioned studies, there are additional studies on brain correlates of EA Scales on the parent–child relationship that are worth noting. First, Schneider-Hassloff, et al. [72] recruited 4–6-year-old children and their mothers, to investigate whether EA is associated with actual behavioral and electrophysiological measures of executive functioning (EF). They administered behavioral EF tasks (head-toes-knees-shoulders task, HTKS, and a delay of gratification task, DoG), and evaluated with the observational EA System. The electrophysiological correlates of EF (go/nogo tasks) were administered and evaluated using event-related potentials (ERP). The authors reported that higher EA nonintrusiveness, as well as higher EA zones scores, were positively associated with the behavioral aspects of EF (that is, HTKS and DoG), and both maternal structuring and nonintrusiveness, and in some cases child responsiveness, were associated with the electrophysiological correlates of EF (that is, in the go/nogo task) [72]. These findings suggest that parenting qualities are associated with the functionality of neural circuits that are involved in the response inhibition component of EFs.

To better understand the child’s side of emotional availability in the mother–child relationship, Licata, et al. [73] investigated left frontal electroencephalogram (EEG) activation vs. right frontal activity in 14-month-old children, and measured EA when children were 7 months and then again at 50 months old. They reported that a higher left frontal EEG activation (as compared to right activation) at 14 months was associated with higher child involvement at 50 months, even when controlling for earlier maternal sensitivity, child responsiveness, and child involvement at 7 months, as well as these EA variables at 50 months. This result implies that child involvement may be dependent on biological factors within the child, and suggest that left frontal asymmetry is related to a child taking the initiative and proactive tendencies in the mother–child relationship. There was no correlation between child responsiveness and frontal EEG activation. Instead, child responsiveness was more closely associated with the mother’s sensitivity [73].

To fully capture all six EA Scales’ relations to different infant emotion cues, Killeen and Teti [74] examined mother’s frontal EEG asymmetry at rest and during emotion-stimulated (joy, anger/distress, and neutral interest) and free-play videos of their infants. Specifically, the researchers recruited 27 right-handed mothers and their 5–8-month-old infants, and recorded infant emotion and mother–infant free-play videos. The free-play videos were used to code for mother–infant EA Scales (maternal sensitivity, maternal structuring, maternal nonintrusiveness, maternal nonhostility, child responsiveness, and child involvement). It was found that maternal frontal EEG asymmetry at rest and during infant emotion videos were not related to mother–infant EA in response to emotional-stimulated states and infant emotion videos, respectively. However, in response to infant anger/distress videos, maternal sensitivity and structuring were related to a shift towards greater relative right frontal activation [74]. The results imply that maternal in-the-moment empathetic response to the infant was related to mother–infant EA.

Moreover, a final study by Taylor-Colls and Pasco Fearon [75] linked parental sensitivity and infant’s neural responses, measured by event-related potentials (ERP), which is an electrophysiological response to a stimulus. Specificity, 40 healthy mothers and their 7-month-old infants were invited to participate, and their interactions were coded using EA Scales (maternal sensitivity, maternal structuring, maternal nonintrusiveness, and maternal nonhostility) during a free play. These maternal dimensions were standardized and summed to form an EA composite. Although the authors seemed to refer to this composite as sensitivity, it is in fact a composite of all the adult scales. Further, given that they used the 4th edition of the system, these are measured on the same metric and contribute equal amounts. The authors found that those with greater maternal EA had increased amplitudes to positive facial expressions, relative to fearful and neutral expressions. The authors argued that maternal responses to the child’s cues contribute to the child’s brain development, and are crucial for their social development and adaptation [75].

### 3.4. Theoretical Framework

**Figure 2:**
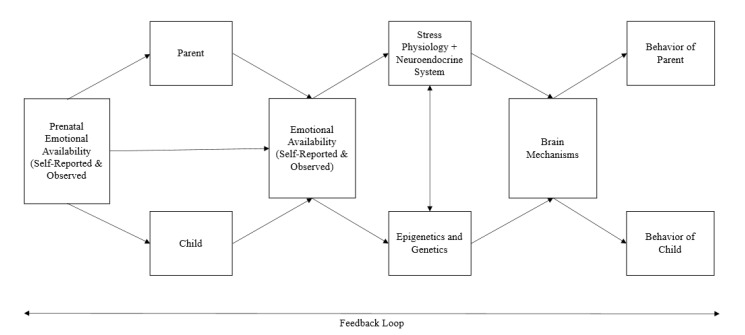
Emotional availability theoretical model.

Pre-EA and postnatal EA, stress physiology, the neuroendocrine system, genetics and epigenetics, brain mechanisms, and behavior bidirectionally interact in ways that are not yet fully understood. For example, past research has shown that changes in parent oxytocin levels are associated with respective changes in emotionally available parenting, and specific parenting emotional availability behaviors lead to changes in oxytocin levels, further emphasizing the transactional nature of the neuroendocrine system [57]. Previous literature is aligned with our theory that specific environmental experiences and parent/child behavioral practices have the potential to influence the brain structure and function in a complex feedback loop, and are thought to be shaped by genetic/epigenetic co-evolutionary processes [26]. With the goal of conceptualizing a theory that integrates these complex concepts [76,77,78], we propose the following theoretical model, presented in Figure 2. In this model, prenatal EA (be it self-reported or observed) is linked with the EA of parent and child postnatally (be it observed or self-reported) and with stress physiology, neuroendocrine system, genetics and epigenetics, and brain mechanisms, and behavior. Each of the included constructs are associated, in multiple ways, with varying strength and direction of associations; however, we have chosen to omit illustrating these interactions for simplicity, and instead have illustrated these dynamic processes through the inclusion of a feedback loop bidirectional arrow. It is important to note that these constructs may influence each other bidirectionally with feedback interactions.

## 4. Discussion

After a general overview of parenting and stress physiology and the neuroendocrine system, genetics and epigenetics, as well as brain mechanisms, we have focused on EA as a worthy area of work in affective neuroscience, with projection that it can bring a consistent framework and measurement specificity. Studies appear to use different measures of sensitivity, and yet refer to them all as sensitivity. Even with the use of the EA System, investigators sometimes hone in on only one or two qualities, rather than take advantage of the entire framework. Particularly noteworthy is how many investigators have reduced EA to ‘sensitivity’ to the neglect of other qualities. Particularly striking is that few studies have focused on the construct of ‘structuring’, even though this is a construct that is closely aligned with scaffolding and teaching, which are qualities that are likely to enhance the brain development of children. In the larger EA literature, adult structuring appears to be a very important quality, and one that is very amenable to intervention efforts [30].

EA is a particularly beneficial measure in brain sciences, due to its utilization of both the parental and child perspectives. Overall, the EA framework’s emphasis on both the parental and the child’s side of the relationship, be it with the EA Scales, EA zones, or EA-SR, is especially promising when investigating how stress physiology and the neuroendocrine system, genetics/epigenetics, may be associated with adults’ as well as children’s brain development, and intergenerational transmission of specific genetic and neurobiological markers. With the exception of Licata and colleagues, few studies have examined the child side of EA with an a priori focus [64]. Including the child’s side of EA would be important, given its links with attachment security [30]. 

Research into prenatal and early life experiences, when the brain is highly malleable and epigenetic changes may occur, is also an important area for future research, in order to better identify potentially modifiable behavioral, genetic, and environmental factors, to promote supportive, quality parent–child relationships during these years. The development of the prenatal EA construct and measurement [34] provides an avenue to investigate parent–child relationships (using an observational lens) from the prenatal period to adolescence [30]. Work on imagined scenarios of conflict and pleasurable interactions [7] with one’s child (even before birth) could open up new areas for intervention and prevention.

Not only can the findings from studies such as the one by Neukel, Bertsch, Fuchs, Zietlow, Reck, Moehler, Brunner, Bermpohl and Herpertz [68] open new areas for intervention and prevention, but they may also provide support for already current parent intervention and prevention groups. If a provider knows a mother has experienced early life maltreatment (ELM), and knows how this life experience may impact the ability of the mother to accurately respond to the cues of her child, the mother may be referred to parent groups that foster sensitivity and responsiveness skills for pleasant mother–child interactions, in addition to negative or challenging interactions. It would be interesting to measure the brain mechanisms and EA of an ELM mother and her child pre- and post-participation in parenting groups that are focused on emotion regulation and building secure attachment. The literature emphasis on child maltreatment-exposed mothers, and the impact on their brain functioning or mechanisms, highlights the need to create and provide support to this population through prevention and intervention efforts.

Moreover, although the dyad can include mothers and fathers, developmental research has only recently begun to study the father–child relationship. Increased incorporation of the father figure has rarely been conducted in this area of inquiry [13], and would provide valuable insights into the similarities and differences between parent figures’ EA and influences on the body.

It may also be interesting to measure the brain’s response to free-play interactions between a mother, father, and child, at the same time. This would provide valuable insights into how the biological response of both parents, who have had similar experiences or relationships to the same child, may differ in the same interactions, and how this may correlate with their EA.

Considering the recent and lasting global impacts of COVID-19, and the heightened stress families have endured, and knowing what we know about the association between stress physiology and EA, it would be interesting and important to study the EA of families who have been most impacted by the virus. Studying children’s cortisol levels who have been significantly impacted by COVID-19-linked stressors (parents’ loss of job, financial instability, death of a parent or close family member) may inform future prevention and intervention efforts focused on improving the EA and quality of relationship between the parent and child.

Finally, it is important to move beyond the parents, to include teachers and child care professionals. Furthering our understanding of stress physiology (salivary and hair cortisol) in these professionals, particularly those working in low-income contexts, will be important. Offering prevention and intervention programs will be key.

## 5. Conclusions and Some Limitations

Utilizing EA to obtain a more comprehensive understanding of the parent–child relationship would provide more specificity in behavioral and affective neuroscience. As the EA field grows in relation to the interconnections of stress and the neuroendocrine system, genetics, epigenetics, and the brain, it is important to utilize all of the EA Scales dimensions (sensitivity, structuring, nonintrusiveness, nonhostility, child responsiveness, and child involvement), as well as EA zones on emotional attachment, to provide the most comprehensive understanding of the strength and direction of the relations between the different constructs. The incorporation of EA into research would provide information that is essential for informing targeted, preventative interventions, to promote healthy parent–child relationships in the present and future. However, more research using a variety of observational measures would also be useful, as for example, molecular indicators, such as synchronous interactions, or frequency counts of behaviors, which the EA System does not offer. Thus, we encourage more observational work including both global as well as molecular measures that can be useful in affective neuroscience, with EA being the global measure that can provide consistency, and with the inclusion of other measures that are more fine-grained rather than holistic. Further, more studies including attachment measures would provide an important focus to this line of work. Table 1, Table 2 and Table 3 show data extraction in detail.

## Figures and Tables

**Table 1:** Characteristics of studies included in the review with a stress physiology and neuroendocrine focus.

Citation Number	Article	Target Population			Methods			Results
		Age	Sample Size	Sample Characteristics	EA Measure	Additional Variables	Evidence of Reliability	
[47]	Kertes et al. (2009)	Child age: M = 3.97 years, SD = 0.48	274 parent-child dyads	Family income: <USD 25,000 to >USD 200,000 (M = in the USD 76–100,000 range) Parent education: range from high school/GED to professional school/doctorate (median education = bachelor’s degree) 83% of children were white, non-Hispanic	Quality of parental interaction: 30-min videotaped parent–child interactions using the emotional availability scales (EAS; Biringen, Robinson, and Emde, 1998). The four parent scales (sensitivity, structuring, nonintrusiveness, and nonhostility) on the EAS were scored; the 4 measures combined into factor score parenting quality	Three salivary cortisol samples; nonsocial and social inhibition	Intraclass correlation coefficients (ICC) > 0.80	Nonsocial and social inhibition could be distinguished; that associations with cortisol response were stressor specific; parenting quality buffered cortisol elevations for extremely socially inhibited children, but not nonsocially inhibited children
[48]	Rickmeyer, Lebiger, & Leusinger-Bhleber (2017)	Mother age: M = 38.77 years	24 mother-child dyads	Infants with an immigrant background, “hard to reach” mothers who had not been integrated into Germany yet; mothers from Turkey and Eastern European countries, African countries, and others	30-min free play session, was assessed with the EA scales, one and a half months before the first cortisol assessment	Hair collection for cortisol (HCC) assessments	4 raters trained and certified, interrater reliability, ICC = 0.942–0.996	Children’s cortisol levels were higher after kindergarten entry than before; lower EA before kindergarten entry associated with a rise in HCC (negative correlation); children with low mother intrusiveness and higher child responsiveness associated with lower cortisol increases
[49]	Senehi et al. (in press)	Child age: 6–36 month old	10 white English speaking mother–child dyads; 29 Spanish speaking mother–child dyads	Participants were recruited from Denver metro area and were enrolled in early head start (EHS)	Quality of the parent–child relationship was assessed via the emotional availability scales	Adverse childhood experiences questionnaire Hair collection for cortisol (HCC) assessments	Interrater reliability, ICC = Not Yet Reported	In relationships with low EA, maternal ACEs (especially with 4 or more ACEs) were significantly associated with increased hair cortisol in their children. The relationship between maternal ACEs and children’s hair cortisol was not significant within the context of relationships with high EA
[50]	Philbrook and Teti (2016)	Child age: 3–9-month-old infants	82 mother–child dyads	Majority white, living with infant’s dad, middle class	Maternal EA during infant bedtime: coded at each age point using the EA scales. Videos set up for one night at 3, 6 and 9 months at family’s home. Coded from the moment camera turned up until 5 min of infant sleep. A composite EA score was calculated	Symptom checklist 90-revised (assesses depressive symptomology); sleep practices questionnaire; infant cortisol using saliva samples	Interrater reliability, ICC = 0.70–0.99	Higher maternal EA at bedtime associated with lower infant cortisol levels; infant’s stress responsivity often affected by mother’s caregiving; greater maternal responsiveness to non-distress infant cues associated with lower infant cortisol levels; more co-sleeping associated with higher cortisol levels
[51]	Ruttle et al. (2011)	Child age: M = 4.5 years, SD = 1.16 Mothers age: M = 30.84 years, SD = 2.87	75 mother–child dyads	Median family income range CND $8430–152,885; M = $43,918); level of mother education ranged from 4 to 18 years (M = 12 years, SD = 2.41)	Mother–child interaction tasks videotaped including the interactive free-play and interference task coded with the EA scales	Salivary cortisol was sampled three times from mother and child across a home visit	Interrater reliability, ICC = 0.84–0.99	Mother–child dyads demonstrate attunement of HPA axis activity; attunement prominent during times of increased challenge; factors associated with behavioral sensitivity may influence attunement within the dyad
[52]	Tarullo et al. (2020)	Child age: 12-month-old infants (86 infants) and 3.5-year-old children (87 children)	173 parent–child dyads (159 mothers, 14 fathers)	Participants were from the greater Boston metropolitan area	12-min videotaped parent–child interaction that included 5-min free play, 5-min structured play, and 2-min clean-up. Only the sensitivity subscale was coded	Hair cortisol concentration (HCC); diurnal salivary cortisol sampled 3 different nonconsecutive times; SES multidimensional assessment (income-to-needs ratio, confusion, hubbub, and order scale, neighborhood organization and affiliation scale-revised, household food insecurity access)	Intraclass correlation coefficients (ICC) for sensitivity subscale infant group ICC = 0.97 3.5-year-old group ICC = 0.81	Parent education was predictive of infant and child HCC. Neighborhood risk predicted infant HCC. Household chaos was related to bedtime salivary cortisol concentration (SCC). Parental sensitivity did not predict or mediate relations between SES and cortisol levels. Higher child HCC was correlated with greater food insecurity for both the infant and 3.5-year-old child group.
[53]	Gilisen et al. (2008)	Group 1 at 4 years old: child age: M = 3.8 years, SD = 0.3 Mothers age: M = 35.9 years, SD = 3.9 Fathers age: M = 38.3 years, SD = 5.2 Group 2 at 7 years-old: Child age: M = 7.4 years, SD = 0.3 Mothers age: M = 38.8 years, SD = 3.4 Fathers age: M = 41.1 years, SD = 4.7	Group 1 at 4 years old: 78 parent–child dyads Group 2 at 7 years old: 92 parent–child dyads (the first-born children of twin pairs)	All participants were born in the Netherlands. Mothers of the 4 year olds had completed 15.9 years (*SD* = 3.9) of education and the fathers had completed 16.3 years (*SD* = 3.7)	10-min free play episode of mother and 4 year old coded using the emotional availability scales	Ambulatory monitoring system to measure skin conductance; parasympathetic influence measured with electrocardiogram (ECG) electrodes; children’s behavior questionnaire (CBQ) to measure child temperamental fearfulness	Interrater reliability, ICC = 0.74–0.87	4 and 7 year olds both responded with increases in skin conductance (sympathetic activation) and decreases in heart rate variability (parasympathetic withdrawal) to the fear-inducing film clip. More fearful children were more susceptible to the quality of their relationships with their mothers than less fearful children, irrespective of their ages.
[54]	Van der Pol et al. (2019)	Child age: M = 4 years, SD = 0.1 Mothers age: M = 35.9 years, SD = 3.9 Fathers age: M = 39.3, SD = 4.7	159 father–child (second born) dyads	The majority of fathers had finished academic or higher vocational schooling (75%); were married or had a registered partnership or cohabitation agreement with the mother of the target child (92%)	Each dyad received a bag with toys and was invited to free play for 8 minutes; coded with the fourth edition of the emotional availability scales	Testosterone concentrations from fathers’ salivary samples; computerized go/nogo task used to measure self-control	Interrater reliability, ICC > 0.70	Higher father basal testosterone levels in the evening were related to less respect for child autonomy (only in fathers with low self-control). Higher father basal testosterone levels in the evening was related to more sensitive parenting (only in fathers with high self-control)
[55]	MacKinnon et al. 2018	Mothers age: M = 35.56 years, SD = 4.36	189 pregnant women	91.0% of mothers reported being married or living with their partner, 3.7% became single, divorced, or widowed since participation in the original study	Five minutes of free play with toys was filmed at follow-up at 7–9 weeks postpartum and coded using the emotional availability scales	Meins and Fernyhough’s (2015) procedure for interactional measures of mind-mindedness in the first year of life; reading the mind in the eyes test for theory of mind; maternal speech transcribed verbatim then identified mind-related comments	Interrater reliability, for all scales (ICCs = 0.51–0.71), except for nonhostility (ICC = 0.38)	Mothers’ reading the mind in the eyes test performance at 7–9 weeks postpartum was associated with more structuring and less intrusive maternal behavior at 2–3 years postpartum, while their tendency to use mind-related comments at 7–9 weeks postpartum was associated with greater sensitivity 2–3 years postpartum
[57]	Naber et al. (2010)	Child age: M = 33.8 months, SD = 11.8 Fathers age: M = 37.9 years, SD = 3.80	17 father–toddler dyads	Participants were all healthy volunteers with at least one child between 1.5 and 5 years of age	2 observed home play sessions of 15 min each coded using the emotional availability scales	single dose of 24 IU oxytocin nasal spray or placebo nasal spray	Interrater reliability average, ICC = 0.95	In the oxytocin condition fathers were more stimulating of their child’s exploration than in the placebo condition, and they tended to show less hostility

**Table 2:** Characteristics of the studies included in the review with a genetics and epigenetics focus.

Citation Number	Article	Target Population			Methods			Results
		Age	Sample Size	Sample Characteristics	EA Measure	Additional Variables	Evidence of Reliability	
[58]	Reichl et al. (2019)	Child age: M = 8.02 years, SD = 1.57 Mothers age: M = 39.28 years, SD = 5.71	193 mother–child dyads	Mothers excluded if they had neurological diseases, severe physical or mental disabilities, or if met criteria for an emotional-unstable, anxious-avoidant or antisocial personality disorder. Children excluded if they met the criteria for an autistic disorder or in case of an intelligence score below 70.	Maternal sensitivity assessed with the sensitivity scale of the emotional availability (EA) scales (Biringen 2008). During two situations (free play; dealing with a hardly solvable puzzle task), mothers were asked to interact with their child without the attendance of any other person.	Analyzed three polymorphisms (rs53576, rs1042778, rs2254298) of the OXTR gene and plasma oxytocin	Inter-rater reliability, ICC ≥ 0.81	Of the three analyzed polymorphisms (rs53576, rs1042778, rs2254298) of the OXTR gene and plasma oxytocin, only the rs53576 was associated with mothers’ parenting behavior, specifically with maternal sensitivity; rs2254298 significantly moderated relations between mothers’ experiences of childhood adversity and parenting behavior; significant relations for mothers homozygous for the G allele; G allele of the rs2254298 was related to increased plasma oxytocin levels
[59]	Lecompte et al. (2021)	Mother age: ≥18 years	16 mother–child dyads	Gestational weeks: 12–14 weeks gestation and pregnant with a single baby	Five minutes of free play with parent and child: filmed and coded with the emotional availability scales	Separation–reunion procedure for preschool-age children; buccal swab for children’s DNA methylation analyses using the Oragene TMOG-250 collection kit; child methylation data at the follow-up time-point from the the OXTR exon 3 genomic region area	Interrater reliability, ICC = 0.51–0.71, sensitivity (.71), structuring (0.54).	Lower maternal sensitivity associated with more controlling caregiving behaviors; less maternal structuring associated with more controlling punitive behaviors; hypomethylation of the OXTR gene associated with greater maternal structuring behaviors and with more child controlling caregiving behaviors; no interaction effect found of OXTR gene as a moderator in the association between interactive behaviors and child controlling behaviors
[60]	Lewis et al. (2020)	Child age: M = 8.5 years, SD = 0.45	N = 96 sub-sample of monozygotic twins	Monozygotic twins: 51% male; 50% Non-Hispanic white, 14.6% Hispanic/Latinx, 8.3% African American, 4.2% Asian American)	EA measured with a 28-item, abridged version of the EA self-report. Items were rated on a five-point Likert scale from “almost never” to “almost always” (asked separately for each twin). Higher scores indicated higher mother-reported EA in the parent–child relationship	General health composite using items from the parent-reported MacArthur health and behavior questionnaire (HBQ) (Essex et al., 2002) Buccal cells were collected with Mawi iSWAB DNA collection tubes (Mawi DNA Technologies LLC, Hayward, CA)	Cronbach’s alpha was 0.721 and 0.806 at 1 and 2.5 years, respectively	Parental EA at 1 year old was related to multiple immune gene methylations in monozygotic twins at 8 years of age. Twin pairs with discordant health, compared to pairs with similar health, had more differences in immune gene methylation

**Table 3:** Characteristics of studies included in the review with brain mechanisms focus.

Citation Number	Article	Target Population			Methods			Results
		Age	Sample Size	Sample Characteristics	EA Measure	Additional Variables	Evidence of Reliability	
[63]	Rodrigo et al. (2020)	Child age: Neglectful, M = 2.8 years, SD =1.5 Control group M = 2.1 years, SD = 1.8 Mother age: neglectful M = 29.2 years, SD = 7.0; control M = 33.43 years, SD = 3.4	48 mother–child dyads (25 neglectful and 23 non-neglectful control mothers)	Mothers with history of neglect of a child in the last 12 months, referral recorded by Child Protective Services, and complied with the indicators of the maltreatment classification system for severe neglect	Mother–child free play; EA scales: six subscales of adult and child EA scales	Mini international neuro-psychiatric interview; T1-weighted magnetic resonance imaging with the MPRAGE (magnetization prepared rapid acquisition gradient echo): gray and white matter volumes	Inter-rater reliability: sensitivity (0.94), structuring (0.90), nonhostility (0.92), nonintrusiveness (0.87), responsiveness (0.92), and involvement (0.86)	Smaller gray matter volume in the right insula, anterior/middle cingulate, and right inferior frontal gyrus and less white matter volume in bilateral frontal regions in the neglectful mothers compared to non-neglectful mothers
[64]	León et al. (2021)	Child age: neglectful: M = 2.7 years, SD =1.5; control group M = 2.3 years, SD = 1.9 Mother age: neglectful M = 29.1 years, SD = 7.1; control group M = 33.6, SD = 3.2	45 mother–child dyads (24 neglectful and 21 non-neglectful control mothers)	Mothers with history of neglect of a child in the last 12 months and complied with the indicators of the maltreatment classification system for severe neglect	Mother–child free play; EA scales: six subscales of adult and child EA scales	Mini international neuropsychiatric interview; T1-weighted magnetic resonance imaging with the MPRAGE (magnetization prepared rapid acquisition gradient echo): cortical thickness and surface area	Inter-rater reliability: sensitivity (0.94), structuring (0.90), nonhostility (0.92), nonintrusiveness (0.87), responsiveness (0.92), and involvement (0.86)	Neglectful mothers showed less cortical thickness in the right rostral middle frontal gyrus and a greater surface area in the right lingual and lateral occipital cortices compared to non-neglectful mother; less right rostral middle frontal gyrus thickness, which relates to a lower level of emotional awareness among neglectful mothers
[65]	Rodrigo et al. (2016)	Child age: neglectful group M = 2.5 years Control group M = 2.3 years Mother age: both groups M = 30 years	44 mother–child dyads (2 neglectful and 22 control)	Mothers with history of neglect of a child less than 5 years of age in the last 12 months, referral recorded by Child Protective Services	Mother–child free play; EA scales: six subscales of adult and child EA scales	Mini mental state examination; mini international neuro-psychiatric review; T1-General Electric 3T scanner	Inter-rater reliability: sensitivity (0.94) structuring (0.90) nonintrusiveness (0.87) nonhostility (0.92) responsiveness (0.92) involvement (0.86)	Neglectful mothers, compared to the control, had disruptions in the structural organization of connectors between the occipital lobe and the temporal and frontal lobes: ILF-R and bilaterally the inferior fronto-occipital fasciculi (IFO-R and IFO-L). Neglectful mothers, compared to controls, showed reduced volumes in ILF-R and IFO-L. Positive mother–child interactions were predicted by increased volume in the ILF-R; neglectful mothers had a higher likelihood of exposure to early adversity, higher vulnerability to psychopathologies, and lower cognitive integrity compared to the control
[66]	Olsavsky et al. (2019)	Mother age: 18–40 years	46 mother–infant dyads (28 childhood maltreatment-exposed and 18 non childhood maltreatment-exposed)	Mothers with childhood maltreatment experiences; first-time mothers	15-min mother–infant free play observation; EA scales: maternal sensitivity, maternal structuring, maternal noninstrusiveness, and maternal nonhostility (focused on maternal sensitivity)	Risky family questionnaire; infant face task; functional MRI: amygdala reactivity	ICC = 0.84	Mothers reporting more childhood maltreatment experiences had greater bilateral amygdala reactivity to infant faces compared to mothers who did not experience childhood maltreatment (observation not exhibited when the childhood maltreatment-exposed mothers were shown adult faces)
[67]	Olsavsky et al. (2021)	Mother age: 18–40 years	61 mothers with childhood maltreatment experiences	Mothers with childhood maltreatment experiences; 18–40 years old; one-time mothers	15-min mother–infant behavioral observations; EA scales: focused on maternal sensitivity, maternal noninstrusiveness, and maternal nonhostility	Risky family questionnaire; infant cry paradigm; functional MRI: amygdala reactivity psychophysiological interaction analyses	ICC = 0.84	Mothers reporting more childhood maltreatment experiences had greater bilateral amygdala response to their own infant’s cry compared to other infant’s cry or white noise; mothers with higher amygdala activation may have decreased intrusive behaviors
[68]	Neukel et al. (2017)	Child age: 7–11 years	47 mother–child dyads (22 mothers with a history of physical and/or sexual childhood abuse and 25 without)	Mothers with a history of physical and/or sexual childhood abuse	Mother–child free play; EA scales: six subscales of adult and child EA scales	Childhood experience of care and abuse interview; structured clinical interview for DSM-IV axis I; international personality disorder examination; Hamilton rating scale for depression; functional MRI	Not specified	Mothers with history of physical and/or sexual abuse or neglect showed greater activation in amygdala, insula and hippocampus; showed less functional connectivity between regions of salience and mentalizing network; mothers with history of physical and/or sexual abuse showed higher maternal sensitivity related to greater bilateral insula and amygdala activations to conflictual versus pleasant interactions
[69]	Mielke et al. (2016)	Mother with early life maltreatment (ELM): M = 38.8 years, SD = 6.7 Mother without ELM: M = 39.1 years, SD = 4.5	25 mother–child dyads with ELM and 28 mother-child dyads without ELM	Mothers are absent of any known substance abuse or neurological disease or dementia or severe physical impairments, or any contraindications for MRI measurements	15-min mother–child free play and 6-min problem-solving task; EA scales: maternal sensitivity	Interpersonal reactivity index; structural magnetic resonance imaging with unbiased voxel-based morphometry	Inter-rate reliability: good Cronbach alpha (0.81–0.88)	Mothers with ELM were less sensitive when interacting with their own child, compared to mothers without ELM; for mothers with ELM, maternal sensitivity was positively associated with the volumes of left superior frontal gyrus extending to the superior medial frontal gyrus and middle frontal gyrus, core regions of the cognitive empathy network; maternal sensitivity was negatively associated with the volume of posterior cingulate cortex
[70]	Firk et al. (2018)	Mother age: M = 27 years SD = 5.3	26 mother–infant dyads	Healthy full-term infants, mothers without any genetic syndrome or severe disease	12-min mother–infant free play, EA scales: maternal sensitivity, maternal nonhostility, and maternal nonintrusiveness	Functional MRI; infant cry stimuli	Interrater agreement: maternal sensitivity (0.96), maternal nonhostility (0.94), and maternal nonintrusiveness (0.92)	Higher maternal sensitivity and higher maternal nonhostility were associated with lower amygdala activation during mother–infant interaction; self-distraction decreased subjective emotional intensity and bilateral activations in the amygdala
[71]	Kim et al., (2017)	Mother age: M = 24.41 SD = 5.22	39 mother–child dyads	First-time new mothers in metro Denver areas recruited from midwifery clinics, Women Infant and Children (WIC) and Colorado State Prenatal Plus programs 46% of sample lived in poverty or near poverty (as determined by an income-to-needs ratio <1 or <2)	Mother–child 15-min free-play was observed and coded using the EA 4th edition scales	Beck depression inventory;	Interrater reliability, ICC = 0.713	Socioeconomic disadvantage was associated with neural sensitivity to infant positive and negative emotions. Lower income-to-needs (ITN) ratio was correlated to reduced responses to positive infant faces. There was evidence of elevated amygdala responses related to negative infant faces. Heightened responses to infant faces was associated with mothers’ intrusiveness.
[72]	Schneider-Hassloff, Zwonitzer, Kunster, Mayer, Ziegenhain and Kiefer [72] (2016)	Mother age: M = 39 years SD = 4.0 Child age: M = 58.7 months, SD = 6.6	27 mother–child dyads	Children absent of any known psychiatric or neurological disease or severe developmental delay	20-min mother–child free play; EA scales: six subscales of adult and child EA scales and EA zones	Head-toes-knees-shoulders task; delay of gratification task; strengths and difficulties questionnaire; colored progressive matrices task	Inter-rater reliability: good (ICC > 0.89) for adult structuring, adult nonintrusiveness, adult nonhostility, child responsiveness, and child involvement; inter-rater reliability: acceptable for adult sensitivity (ICC = 0.67), inter-rater reliability: low for EA CS (ICC = 0.55)	Higher EA nonintrusiveness was associated with the behavioral aspects of executive functioning; maternal structuring and nonintrusiveness were associated with electrophysiological correlates of EF
[73]	Licata et al. (2015)	Child age: accessed at 7, 14 and 50 months Final sample M = 6.95 months, SD = 0.22	Sample of 28 children (15 girls)	Mothers diagnosed with postpartum depression and/or anxiety disorders	EA scales, 10-min videotaped interaction, only maternal sensitivity, maternal structuring, child responsiveness and child involvement subscales were used	Vulnerable attachment style questionnaire; structured clinical interview; theory of mind	At 7 months of child’s age: inter-rater reliability maternal sensitivity (0.89), child responsiveness (0.88), and child involvement (0.78) At 50 months of child’s age: maternal sensitivity (0.85), child responsiveness (0.84), and child involvement (0.89)	Low maternal attachment style insecurity and high theory of mind skills predict maternal EA sensitivity
[74]	Killeen & Teti (2012)	Mother age: M = 30.7 yearsInfant age: M = 6.94 months	27 mother–infant dyads	Right-handed mothers	30-min mother–infant free play; EA scales: maternal sensitivity, maternal structuring, maternal nonintrusiveness, maternal nonhostility, child responsiveness, and child involvement	Electroencephalogram; infant emotion videos; SCL-90-R depression and anxiety subscales; maternal self-reported emotional experience	ICC: 0.668–0.738 for maternal sensitivity, maternal structuring, maternal nonintrusiveness, child responsiveness, and child involvement, 0.411 for maternal nonhostility	EA or mother-reported emotional experience in response to infant emotion cues was not related to the greater relative right frontal activity at rest; greater mother–infant EA was associated with a shift toward greater relative right frontal activation in response to infant emotion cues
[75]	Taylor-Colls & Fearon (2015)	Child age: 7 month old (24 males)	40 mother-child dyads	Healthy infants’ absence of low birth weight or premature birth	3-min mother–child free play; EA scales: maternal sensitivity, maternal structuring, maternal noninstrusiveness, and maternal nonhostility	Event-related potentials; infant behavior questionnaire	Inter-rater reliability: reasonable (ICC = 0.71–0.75)	Higher maternal sensitivity was related to infants’ greater amplitudes to positive facial expressions, relative to fearful and neutral expressions.
